# *DDX23* gain-of-function linked to autism spectrum disorder with intellectual disability, motor delay, and seizure

**DOI:** 10.1016/j.gimo.2026.104484

**Published:** 2026-07-05

**Authors:** Ranjan K. Sahu, Lynne M. Bird, Alexa R. Geltzeiler, Pradeep Vasudevan, Marcelo Vargas, Beau Crabb, Jessica Ruedy, Wendy K. Chung, Hyung-lok Chung

**Affiliations:** 1 Department of Neurology, Houston Methodist Research Institute, Houston, TX; 2 Department of Pediatrics, University of California San Diego/Rady Children’s Hospital, San Diego, CA; 3 Department of Pediatrics, Boston Children’s Hospital, Boston, MA; 4 Department of Clinical Genetics, University Hospitals of Leicester NHS Trust, Leicester, UK; 5 Department of Medical Genetics, Gillette Children’s Hospital, Minneapolis, MN; 6 Department of Pediatrics, Harvard Medical School, Boston, MA; 7 Department of Neurology, Weill Cornell Medical College, New York, NY

**Keywords:** DEAD-box helicase, Developmental delay, Gain-of-function, Intellectual disability, Missense variants

## Abstract

**Purpose:**

DDX23, a member of the DEAD-box RNA helicase superfamily, is crucial for RNA processing and translation initiation during gametogenesis and cell division. This study aims to determine the disease-causing potential and clinical relevance of rare de novo *DDX23* missense variants identified in individuals with neurodevelopmental and cardiopulmonary anomalies.

**Methods:**

Clinical and genetic analyses were performed on 3 individuals presenting with developmental delay or intellectual disability, facial dysmorphisms, brain structural abnormalities, and cardiopulmonary defects. Exome sequencing identified rare de novo monoallelic missense variants at p.Arg528–p.(Arg528Cys), p.(Arg528His), or p.(Arg528Gln)–in *DDX23*. In silico prediction tools were used to assess the potential functional impact of these variants. To test their disease-causing role in vivo, wild-type and two mutant *DDX23* alleles, p.(Arg528Cys) and p.(Arg528His), were expressed ubiquitously or in a tissue-specific manner in a humanized *Drosophila* model.

**Results:**

All 3 individuals carried a de novo missense variants at p.Arg528–p.(Arg528Cys), p.(Arg528His), or p.(Arg528Gln)–all predicted in silico to be deleterious, with dominant effects. Expression of the p.(Arg528Cys) and p.(Arg528His) alleles in *Drosophila* resulted in severely compromised development and survival, with either ubiquitous or tissue-specific expression leading to a complete loss of progeny. These findings demonstrate a robust functional impact of the variants consistent with a gain-of-function mechanism.

**Conclusion:**

Our study identifies recurrent de novo missense variants in DDX23 as the genetic cause of a neurodevelopmental syndrome. Functional assays in *Drosophila* confirm the disease-causing potential of these variants and support a gain-of-function mechanism for the p.(Arg528Cys) and p.(Arg528His) alleles.

## Introduction

Neurodevelopmental disorders are a diverse spectrum of diseases characterized by abnormal brain development, which disrupts cognition, motor abilities, communication, and behavior. Intellectual disability (ID) and profound autism are associated with rare genetic variations that play roles in the development of the brain and its function by affecting neurogenesis, neural circuits, and synapse function, which are crucial for normal development, cognition, and behavior.

ID and autism have been associated with rare de novo variants in genes and copy number variations. Among the genes implicated are DEAD-box helicases, a family that has a critical role in RNA splicing,^1^, ^2^, ^3^ translation regulation,^4^ and RNA degradation.^5^ Disruptions in splicing factors have been shown to impact neuronal differentiation, synaptic function, and the expression of genes necessary for proper brain connectivity.^6^, ^7^, ^8^, ^9^, ^10^, ^11^ Although DEAD-box helicases have also been implicated in other contexts, such as suppressor of translation of toxic peptides^12^^,^^13^ and innate immune signaling,^14^^,^^15^ this study focuses on DDX23, a core spliceosomal helicase (MIM 612172), whose perturbation is likely to affect neuronal development through impaired pre-mRNA processing. DDX23 is highly conserved from yeast to humans and has a well-established role in RNA surveillance. Genetic variants in *DDX23* have been implicated in severe neurodevelopmental disorders,^6^^,^^14^ including autism and seizures.

A study reported 9 unrelated individuals with de novo DDX23 missense variants, all located within conserved RecA-like helicase domains. One of the most informative individuals (patient #2) presented with global developmental delay (DD), with seizures beginning at age 5 followed by language loss, severe myopia, corpus callosum abnormalities, delayed motor milestones, and persistent hypotonia.^6^ However, despite this clear clinical association, none of these human variants had been functionally assessed in an animal model, limiting the mechanistic understanding of their disease-causing role. In this study, we report 3 unrelated individuals who all carry a heterozygous de novo p.(Arg528Cys) (c.1582C>T) (Supplemental Dataset 1), p.(Arg528His) (c.1583G>A) (Supplemental Dataset 2), or p.(Arg528Gln) (c.1583_1584delGTinsAA) allele in DDX23 (NM_004818.3). All individuals have DD, ID, and/or autism.

To elucidate the disease-causing potential of the *DDX23* variants, we expressed these variants in *Drosophila*, a well-established model organism for studying conserved genetic pathways in neurodevelopment. The *Drosophila* survival results support gain of function (GoF) as the disease-causing mechanism for the p.(Arg528His) or p.(Arg528Cys) allele. Our study provides critical experimental evidence using *Drosophila* models, thereby strengthening the causal link between the DDX23 variants and neurodevelopment.

## Materials and Methods

### Identification of patients and genetic analysis

Patients were identified through clinical exome sequencing performed as part of their diagnostic evaluation and were connected through GeneMatcher. Informed consent was obtained from all patients or their legal guardians in accordance with institutional ethical guidelines. Studies were approved by the institutional review board at Boston Children’s Hospital. Data from the DECIPHER community were also included in this study.

Sanger sequencing and all genomic analyses were performed using the human reference genome GRCh38 (RefSeq assembly GCF_000001405.39; chromosome 12: NC_000012.12), obtained from the National Center for Biotechnology Information database. All variants were annotated based on the *DDX23* RefSeq transcript NM_004818.3. Orthologous information was obtained from the MARRVEL database. All variants were reported according to the Human Genome Variation Society nomenclature at the protein (p.), coding DNA (c.), and genomic (g.) levels as follows:

• p.(Arg528Cys): NM_004818.3:c.1582C>T; NC_000012.12:g.48833498G>A
• p.(Arg528His): NM_004818.3:c.1583G>A; NC_000012.12:g.48833497C>T
• p.(Arg528Gln): NM_004818.3:c.1583_1584delGTinsAA; NC_000012.12:g.48833496_48833497ACinsTT

### Fly husbandry and fly stocks

Flies were reared at 24 ± 1 °C, and in vials containing standard cornmeal and molasses medium. All transgenic fly lines used for this investigation were either generated or acquired from the Bloomington Drosophila Stock Center.

### Generation of humanized UAS-DDX23 fly lines

In this study, we used the *DDX23* complementary DNA encoding the full-length gene as a template to generate the reference and variant alleles of *DDX23*. These alleles were generated via site-directed mutagenesis and were cloned under the upstream activating sequence promoter present in the pUAST-attB cassette. To ensure comparable expression levels, these transgenes were subsequently injected into *y*^*1*^*,w*^*1*^ embryos having the attP2 site on the third chromosome. Positive transformants were identified by screening progeny for the presence of red eyes and were balanced with a third chromosome balancer.

### Drosophila viability assay

Desired flies were reared at 24 ± 1 °C, and viability was determined by comparing the number of adults of the desired genotype to the number of eclosed balancer-carrying progeny relative to the expected Mendelian ratios. Viability was analyzed using 3 independent biological replicates; for each replicate, approximately 80 to 150 flies were scored per genotype.

### qPCR and western blotting

Total RNA was isolated from larvae of the desired genotypes and checked for its purity and concentration. For quantitative polymerase chain reaction, 0.5 μg of RNA per replicate was analyzed in triplicate with RP49 for normalization. For western blot, protein lysates were prepared from the desired larval tissues and were separated on a polyacrylamide gel, transferred to a polyvinylidene fluoride membrane, and probed with *hDDX23* (ab70459-Abcam) and β-tubulin (DSHB-E7) antibodies.

### Data analysis

All statistical analyses in this study were conducted using GraphPad Prism 10 (GraphPad Software). All experiments were performed with at least 3 biological replicates. Data are presented as the mean ± SEM. Pairwise comparisons were performed using 2-tailed unpaired *t* tests. A cutoff point of *P* < .05 was established for the statistical level of significance.

## Results

### Identification of DDX23 variants and their associated phenotypes

All patients had de novo heterozygous missense variants at p.(Arg528) in *DDX23* (1 p.(Arg528His), 1 p.(Arg528Cys), and 1 p.(Arg528Gln)) identified on exome sequencing (Table 1). Case #2 was previously published,^6^ and case #3 was identified in the DECIPHER database.^16^ Their current ages range from 3 to 27 years. All individuals had DD that was recognized in early childhood, and 2 of the 3 individuals had features identified prenatally, including intrauterine growth restriction or an echogenic cardiac focus. All children demonstrated global DD. Two of the 3 children were nonverbal, and 1 had autism. The oldest child had medically intractable seizures treated with 4 antiseizure medications. He had 2 periods of regression in association with poorly controlled seizures and did not recover his milestones completely, even when seizures were subsequently controlled. Two children had undergone brain magnetic resonance imaging, both of which demonstrated decreased cerebral and white matter volumes. All 3 had dysmorphic craniofacial features, including temporal flattening, a prominent forehead, plagiocephaly, widely spaced eyes, large ears, and a short, broad nasal bridge. The 3-year-old patient was G-tube dependent, had a history of hypertension and bilateral sensorineural hearing loss treated with hearing aids, and a history of trivial bilateral pulmonary artery stenosis that had resolved with age.

**Table 1:** Summary of genetic and behavioral aberrations in individuals with DDX23 variants and neurological disorders

Individual	1	2^6^	3
Variant	c.1583G>A	c.1582C>T	c.1583_1584delGTinsAA
	p.(Arg528His)	p.(Arg528Cys)	p.(Arg528Gln)
Inheritance	de novo	de novo	de novo
CADD	26.1	32	
Alpha missense	0.9994	0.9997	
gnomAD v4.1.0	1	0	0
Gender and age (years)	M,3	M,13	F,27
Parental history	IUGR, C-section for breech, hypoglycemia after delivery, 36 h in NICU	Echogenic focus, left ventricle	Unknown
Dysmorphic features	Temporal flattening, prominent forehead, plagiocephaly, widely spaced almond shaped eyes, bilateral epicanthal folds, large ears. short broad nasal bridge	Protuberant ears, protruding philtrum with poorly defined ridges, down-turned corners of mouth	Downturned corners of mouth, epicanthus, low anterior hairline, micrognathia, narrow mouth, prominent nasal bridge, short foot, short toe, triangular face
Developmental delay	Y	Y	Y
Speech abilities	Absent speech	Absent speech	Speaks at a 2 y/o level
Intellectual disabilities	Unknown	Y	Y, severe
Hypotonia	Y	Y	Y, as an infant
Seizures	N	Y, intractable, requiring 4 medications (Epidiolex, Onfi, Keppra, phenytoin)	N
Seizure type/EEG results	NA	Infantile spasms Focal seizures (onset 5 y) Atonic seizures (onset 9 y) Seizures present on video EEG	NA
Seizure frequency	NA	Variable, ranging from many per day to absence clusters every 2 weeks	NA
Autism	N	Y	N
ADD/ADHD	N	N	N
Other neurological/neuromuscular disorder(s)	N	Slow ambulation	Wide based, stiff gait
Brain MRI/CT	Brain MRI: Diffuse mild to moderate cerebral brain parenchymal volume loss	At 11 mo: Decreased white matter volume. Bilateral small choroid plexus cysts. At 5 y, 6 mo: Subtle asymmetric volume loss of the left frontal lobe. Mega cisterna magna. At 11 y: Prominent cisterna magna. Mild thinning of the anterior corpus callosum.	Not performed
Immunology problems	Respiratory illnesses and recurrent ear infections in early childhood	N	N
Self-care abilities (eg, dressing, feeding)	Needs significant assistance for all self-care	Not toilet trained, some ability to feed self, but dependent for most care	Needs help with dressing, feeding, and toileting
Other significant medical problems	Oropharngeal dysphagia, nail hypoplasia, nasolacrimal duct stenosis	Unknown	N
Other genetic testing results	Negative CGH SNP Array	mtDNA sequencing and deletion analysis negative	N

*ADD*, attention-deficit disorder; *ADHD*, attention-deficit/hyperactivity disorder; *CADD*, Combined Annotation Dependent Depletion; *CGH*, comparative genomic hybridization; *CT*, computed tomography; *EEG*, electroencephalography; *F*, female; *gnomAD*, Genome Aggregation Database; *IUGR*, intrauterine growth restriction; *M*, male; *MRI*, magnetic resonance imaging; *mtDNA*, mitochondrial DNA; *N*, no; *NA*, not applicable; *NICU*, neonatal intensive care unit; *SNP*, single-nucleotide polymorphism; *Y*, yes; *y/o*, years old.

### DDX23 variants are predicted to be dominant

**Figure 1:**
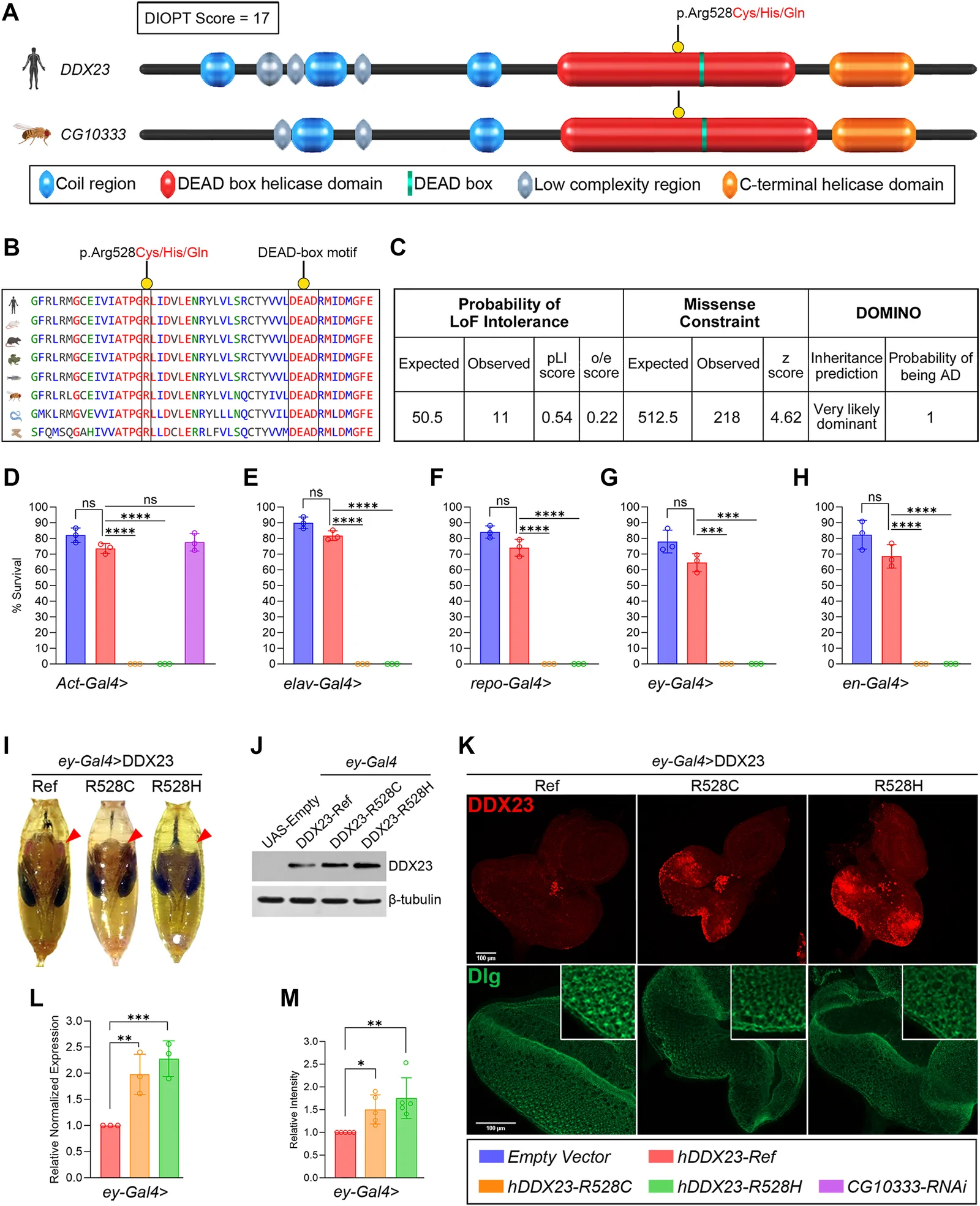
**DEAD-box helicase domains of DDX23 are ultraconserved and the expression of hDDX23 variants demonstrates a gain-of-function mechanism. (**A) Protein domain organization of human DDX23 and its fly ortholog CG10333 (with a DRSC Integrative Ortholog Prediction Tool score of 17) and positions of its variants. The variants are indicated with yellow circles outlined in black. Schematic drawing of the DDX23 enzyme showing the position of the p.(Arg528Cys), and p.(Arg528His), and p.(Arg528Gln) variants within the DEAD-box helicase domain, which is crucial for the catalytic activity of DDX23. The p.Ala706Val variant positioned at the C-terminal DEAD-box helicase domain of DDX23. (B) Protein sequence alignment of human DDX23 with other species including *Drosophila*. The variants are highlighted in black-outlined regions. (C) The *DDX23* variants are predicted to be dominant with a score of 1 for ‘‘probability of being autosomal dominant” by DOMINO. **(**D) Ubiquitous (E) Neuronal (F) Glial (G) Eye specific and (H) Wing specific expression of the hDDX23 p.(Arg528Cys) or p.(Arg528His) results in complete loss of viability. (I) Dead pupa showing complete loss of eyes (indicated by red arrowheads) when these variants were expressed in the eye. (J) Representative Western blot of ey-Gal4 driven eye imaginal discs expressing DDX23 reference or variant (R528C and R528H). β-tubulin serves as loading control. (K) Immunostaining of larval eye discs stained for DDX23 (red) and Dlg (green) to visualize photoreceptor organization. Both variants exhibit higher DDX23 levels with R528H showing slightly greater disruption of photoreceptor arrangement compared to R528C. (L) Quantification of Western blot signals showing ∼2.0- and ∼2.3-fold higher DDX23 protein levels in R528C and R528H variants, respectively, compared to reference. (M) Quantification of immunostaining intensity shows ∼1.5- and ∼1.8-fold increases for R528C and R528H, respectively. Scale bars: 100 μm. Data represent mean ± SEM from at least 3 independent biological replicates.

The MARRVEL online resource (https://marrvel.org) provided the data for the human *DDX23* gene and its orthologs in different model organisms.^17^ With a loss-of-function (LoF) variation observed/expected (o/e) ratio of 0.22 and a probability of being LoF intolerant score of 0.54, *DDX23* is moderately intolerant to its LoF, and the variants in *DDX23* are 100% likely to be autosomal dominant (Figure 1C), as predicted by DOMINO.^18^ With a missense variant o/e ratio of 0.425 and a *z* score of 4.62, *DDX23* is missense constrained according to gnomAD (Genome Aggregation Database).^19^ The region surrounding amino acid 528 is functionally important and moderately constrained (*z* = 3.21), as it harbors fewer genetic variants than anticipated in the normal population. This suggests that the region is under evolutionary pressure to remain constrained, implying its functional importance. Consequently, genetic alterations in this region are likely to have deleterious effects.

As the fly homolog of *DDX23*, CG10333 has an overall 63.8% identity and 76.8% similarity at the protein level (Figure 1A and B). A highly conserved DEXDc (ATPase) domain with 85% similarity and the HELICc domain with 95% similarity are shared by the human and *Drosophila* proteins (Figure 1A). The arginine residue at position 528 of *DDX23* remains well conserved throughout evolution, spanning from lower to higher eukaryotes. All variants of this residue (p.(Arg528Cys), p.(Arg528His), and p.(Arg528Gln)) are integral parts of the DEAD-box helicase (Figure 1A).

### DDX23 variants show elevated protein levels and photoreceptor disorganization

To assess the effect of disease-associated DDX23 variants on protein expression, we performed Western blot analysis on lysates from *ey-GAL4* driven larvae expressing either reference or variant hDDX23. Both the R528C and R528H variants exhibited markedly higher protein levels than the reference, with approximately 2- and 2.3-fold increases, respectively (Figure 1J and L). These results were consistent with the immunohistochemical analysis of larval eye discs, where DDX23 staining revealed elevated protein levels of approximately 1.5-fold for R528C and approximately 1.8-fold for R528H relative to the reference (Figure 1K and M).

In addition to increased DDX23 expression, anti-Dlg staining of eye imaginal discs revealed mild but consistent disruptions in photoreceptor arrangement in both variants (Figure 1K). While the reference discs displayed the expected regular ommatidial patterning, variant-expressing discs showed subtle misalignment of photoreceptors, suggesting that these variants may perturb local structural organization during eye development. Together, these data indicate that the R528C and R528H variants not only elevate DDX23 protein levels but also affect photoreceptor patterning in vivo, providing a potential link between variant expression and neural structural defects.

### DDX23 variants likely function via GoF mechanism

To express the human *DDX23* variants, we generated a *Drosophila* model to investigate the disease-causing potential of the variants in vivo. In a wild-type genetic background, we generated transgenic flies for *DDX23* reference *(UAS-DDX23 Ref)* and 2 missense variants *(UAS-DDX23- p.(Arg528Cys) and p.(Arg528His))*. The variants were expressed using the *UAS-GAL4* system in different cell types, including neuronal *(elav-Gal4)*, glial *(repo-Gal4)*, wing *(en-Gal4),* eye *(ey-Gal4),* and *Actin-Gal4* for their ubiquitous expression, and were assayed for their viability, longevity, climbing ability, and response to vibration shock (bang-sensitivity).

The examination of *DDX23* reference and disease-causing variants in *Drosophila* offers valuable insights into how variations at specific loci can profoundly affect organismal development and survival. Upon the ubiquitous expression of *DDX23* reference and 2 variants, both variants resulted in complete lethality, yielding no viable progeny (Figure 1D-H). The lethality manifests at the embryonic stage when expressed ubiquitously, as indicated by the absence of larvae with the expected genotypes in experimental assays.

The detrimental effect of these 2 variants remained evident when their expression was restricted to brain cells, as indicated by the lack of viable progeny when they were expressed in either neuronal or glial cells (Figure 1E and F). This indicates that the arginine residue at position 528 is crucial, emphasizing that the adverse effect is not tissue-specific but fundamentally linked to the variant itself. This finding corroborates the recognized role of *DDX23* in neurological pathways and suggests that variants at this location hamper critical neurodevelopmental processes.

Expanding the inquiry to tissues such as the wing and eye (when expressed with *en-Gal4* and *ey-Gal4*, respectively) yielded consistent findings. Like previous observations, these 2 variants resulted in complete lethality (Figure 1G and H), with pupal developmental arrest. The pupae were notably lacking eyes and heads (Figure 1I), indicating that the development stopped at the pupal stage with the expression of the mutant alleles in the eye.

The partial downregulation of *Drosophila CG10333* does not significantly impact viability or organ development (Figure 1D), though it remains unclear whether DDX23 has more subtle or context-dependent roles. The expression of the variants causes severe functional disruption, leading to complete lethality and developmental abnormalities. The findings indicate that the variation at this residue is likely to act through a GoF mechanism. This suggests that the variants likely confer a new, harmful function or induce abnormal interactions that disrupt critical pathways involved in cellular differentiation and organogenesis. The stark difference in phenotypic outcomes between loss-of-function knockdown and mutant allele expression highlights the potential role of the variants in pathological GoF mechanism.

## Discussion

We report 3 individuals with DD or ID who have rare variants in *DDX23*, including multiple independent cases affecting amino acid 528. This recurrence of variants at this residue highlights its critical role. The similarity in human phenotypes and our in vivo analysis suggest that these monoallelic *DDX23* variants in the DEAD-box domains lead to a syndromic form of DD or ID associated with dysmorphic features and variably associated with autism, epilepsy, and congenital cardiopulmonary disease.

Variants at amino acid 528 are in proximity to the conserved DEAD motif and likely disrupt the ATP-dependent RNA helicase activity. This disruption may impair RNA processing, including splicing and translation regulation, contributing to the observed neurodevelopmental disorder.

In contrast to the findings of the previous study,^6^ our study expands the number of patients and alleles and provides in vivo experimental evidence using a *Drosophila* model to evaluate the functional consequences of *DDX23* variants. Expression of the human missense variants in neuronal and glial tissues results in severe viability defects, demonstrating that these alleles disrupt nervous system function in vivo, consistent with a pathogenic GoF effect. This is further supported by elevated DDX23 protein levels in variant-expressing larvae. The expression of DDX23 variants in nondispensable organs, such as the wings and eyes, results in complete lethality, highlighting the essential role of DDX23 in proper tissue development. This outcome is consistent with previous findings, which similarly reported strong viability defects for other candidate genes,^20^^,^^21^ suggesting that dysregulation of DDX23 abundance may directly contribute to pathogenicity.

Although partial knockdown of *Drosophila DDX23* does not provide a fully efficient loss-of-function model (Supplemental Figure 1) and therefore cannot establish a clear contrast, the severe phenotypes induced by the human variants, which are absent in knockdown conditions, suggest that these alleles act via a GoF mechanism rather than simple haploinsufficiency.

Our morphological analyses also support the developmental disruption of the variants. Eye imaginal discs expressing human variants show mild alterations in photoreceptor arrangement, pointing to a structural abnormality during neurodevelopment. While these findings indicate disrupted neural patterning, they do not yet establish direct parallels to the patient phenotypes. Further analyses, such as assessment of neuronal morphology, assessment of synapses at the neuromuscular junction, and evaluation of cell proliferation or apoptosis, could reveal early developmental defects and determine the extent to which the *Drosophila* model recapitulates human disease. Transcriptomic profiling (eg, RNA sequencing) and downstream pathway-level analyses represent important priorities for future work, as these approaches could identify disrupted molecular networks and provide deeper mechanistic insight.

This in vivo validation provides a functional framework for understanding variant-specific effects in vivo and establishes a foundation for future therapeutic investigations, including allele-specific RNA interference or an antisense oligonucleotide approach to selectively degrade the variant transcripts.

## Data Availability

All releasable data are provided in the manuscript and supplemental information.

## Ethics Declaration

Written informed consent was obtained from each study participant. As part of the informed consent process before analysis, patients were educated about the significance and limitations of next-generation sequencing diagnostics and the potential for incidental findings. In all analyses, consent was given for the report of incidental findings with therapeutic relevance. All genetic analyses and investigations were performed in accordance with the guidelines of the Declaration of Helsinki and were approved by local institutions.

## Conflict of Interest

The authors declare no conflicts of interest.
