# Supplementary material for: DDX23 gain-of-function linked to autism spectrum disorder with intellectual disability, motor delay, and seizure
Source: Genet Med Open. 2026 Jul 5;4:104484. doi: 10.1016/j.gimo.2026.104484 (PMC13507733; doi:10.1016/j.gimo.2026.104484)
Supplement: Supplementary file 1 [file mmc1.pdf]

Supplementary Figure 1

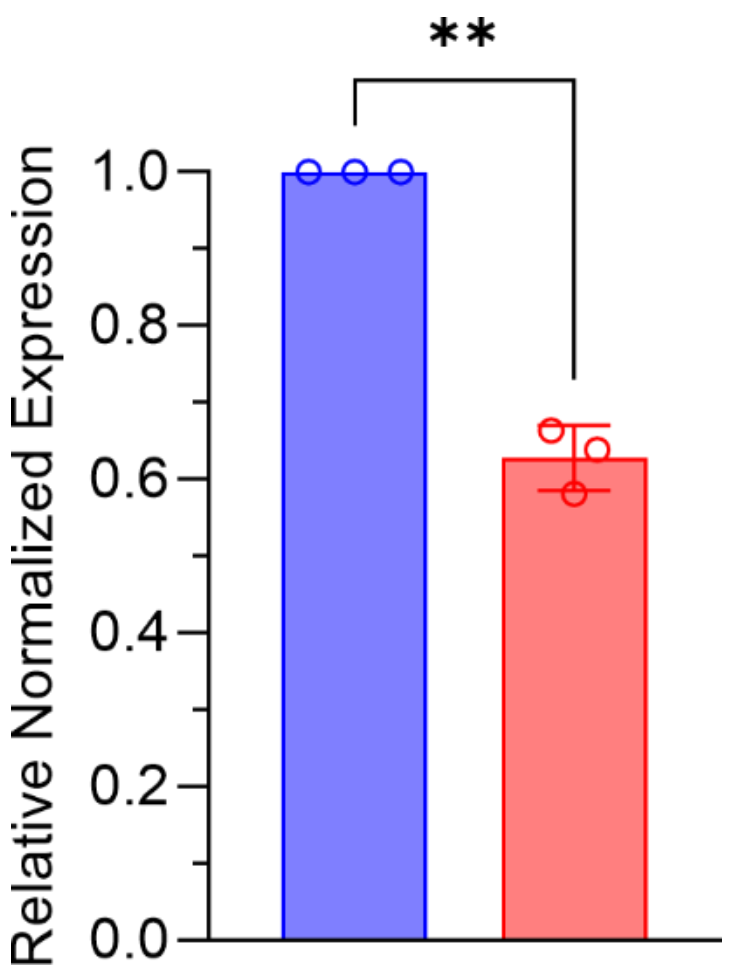

Supplementary Figure 1. Partial knockdown of Drosophila DDX23 (CG10333). qPCR analysis shows ~40% reduction of CG10333 transcript driven by Actin-Gal4. Blue bars: Act>UAS-Empty controls; red bars: Act>CG10333-RNAi. Primers used for qPCR were: CG10333-For GATAGGCAGTTCGATAGGGAGA and CG10333-Rev GACGTTCGGGTGACCTTCG.
