# Supplementary material for: DDX23 gain-of-function linked to autism spectrum disorder with intellectual disability, motor delay, and seizure
Source: Genet Med Open. 2026 Jul 5;4:104484. doi: 10.1016/j.gimo.2026.104484 (PMC13507733; doi:10.1016/j.gimo.2026.104484)
Supplement: Supplementary file 2 [file mmc2.pdf]

[redacted]

GeneDx

REVISED REPORT

DOB
[redacted]/2011

Accession
1590453

Submitter Patient ID(s) [redacted], L16-109R28

Sample
Source: Blood in EDTA
Date Collected: 4/18/2016
Date Received: 4/19/2016

Testing
Date Started: 8/26/2019
Date Reported: 7/18/2022

Provider
Account #: ZB020
KONERSMAN, MD, CHAMINDRA

Test(s)Requested

Diagnostic Testing / XomeDx Reanalysis / Reanalysis of Previously Reviewed Exome Sequence Data

Reason for Corrected Report

This report replaces the original report dated 10/24/2019. The DDX23 gene, formerly a candidate (novel) gene, is now considered a validated gene associated with the phenotype(s) discussed in the gene summary below. The previously reported c.1582 C>T p.(R528C) variant has been reclassified from a variant of uncertain significance to a pathogenic variant. See the revised variant interpretation below. The classification of other reported variants, if present, has not changed, but minor updates to other sections of the report may be included.

Clinical Indication

Individual with global developmental delay, intellectual disability, autism spectrum disorder, severe myopia, hypotonia, epilepsy, white matter volume loss on brain MRI, and mild dysmorphic features

Result: Positive

- Causative variant(s) in disease genes associated with reported phenotype: **POSITIVE**
- Variant(s) in disease genes possibly associated with reported phenotype: **None Identified**
- ACMG Secondary Findings: **None Identified**

Causative Variant(s) in Disease Genes Associated with Reported Phenotype:

Gene	Disease	Mode of Inheritance	Variant	Zygosity	Inherited From	Classification
DDX23	DDX23-related neurodevelopmental disorder	Autosomal Dominant	c.1582 C>T p.(R528C)	Heterozygous	De Novo	Pathogenic Variant

Interpretation

This individual is heterozygous for a pathogenic variant in the DDX23 gene, which is consistent with the reported developmental delay, autism spectrum disorder, and epilepsy. As both parents tested negative for the variant, most likely it arose de novo in the child; however, the possibility of germline mosaicism must be considered.

Recommendation(s)

- Genetic counseling is recommended to discuss the implications of these results.
- Correlation of these findings with the clinical features of this individual is recommended.
- Though neither parent has the pathogenic variant, the possibility of germline mosaicism cannot be excluded and offspring of either

parent may be at risk to inherit this variant.
- If desired, molecular prenatal diagnosis is available for the pathogenic variant.

DDX23

GENE SUMMARY

The DDX23 gene is a member of the DEAD box family of RNA helicases plays a role in the formation of an active spliceosome and pre-mRNA splicing (PMID: 24914973). De novo variants in the DDX23 gene have been reported in a small number of individuals with developmental disorders (PMID: 34050707, Iossifov et al., 2014, Deciphering Developmental Disorders Study). Affected individuals' features include tone abnormalities, developmental delay, facial dysmorphism, autism spectrum disorder, and seizures (PMID: 34050707). Missense variants have been reported to date (PMID: 34050707, HGMD)

For this gene, 100% of the coding region was covered at a minimum of 10x by this test. There is no indication of a multi-exon deletion/duplication involving this gene in the sequencing data

p.(Arg528Cys) (CGT>TGT): c.1582 C>T in exon 13 of the DDX23 gene (NM_004818.2)

The proband's mother (GeneDx #1590466) and father (GeneDx #1590480) do not harbor the p.(R528C) variant in the DDX23 gene.

- De novo variant with confirmed parentage in a patient with global develepmental delay, decreased white matter, corpus callosum abnormalities, and seizures in the published literature, who is likely this same patient (Burns et al., 2021)
- De novo variant with confirmed parentage in a patient from a cohort of individuals undergoing exome sequencing for developmental disorders (Deciphering Developmental Disorders Study, 2017)
- Not observed in large population cohorts (gnomAD)
- In silico analysis, which includes protein predictors and evolutionary conservation, supports a deleterious effect

We interpret this as a Pathogenic Variant.

Additional Analysis Comments

The analysis for the proband includes evaluation of variants that are identified to be de novo (when both parents submitted), compound heterozygous (when both parents submitted), homozygous, heterozygous and X-linked. In addition, analysis takes into consideration family structure, reported phenotype, and provided clinical and/or differential diagnosis. This is a phenotype-driven test of a very large number of genes. Therefore, reported results are focused on pathogenic and likely pathogenic variants in genes related to the phenotype of the proband, while variants of uncertain significance are only rarely reported at our discretion. Sequence variants are classified based on the ACMG/AMP guidelines (Richards et al., 2015). Updates to the classification of a sequence variant may be accessed through ClinVar (www.clinvar.com). Independent analysis of submitted relative samples is not performed unless ordered separately.

ACMG Secondary Findings Limitations

Known or expected pathogenic variants in the genes recommended by the ACMG SF v2.0 are reported for the proband, in accordance with the reporting structure recommended by the ACMG (Kalia et al., 2017; Green et al., 2013); see Appendix 1 for this list of genes (Kalia et al., 2017). The presence or absence of the proband's identified secondary findings is available for relatives who underwent whole exome sequencing or targeted segregation analysis as part of the proband's test. ACMG secondary findings are not reported for relatives that opted out of receiving ACMG secondary findings. Variants that may be present in a relative, but are not present in the proband, would not be detected and therefore are not reported. Only variants that have been previously reported and are a recognized cause of the disorder (known pathogenic; KP) or variants that are previously unreported but are of the type which is expected to cause the disorder (expected pathogenic; EP), as specified in the ACMG recommendations are reported (Kalia et al., 2017; Green et al., 2013; Richards et al., 2015). Known or expected pathogenic variants may be present in a portion of the gene not covered by this test and therefore would not be detected. The absence of reportable secondary findings for any particular gene does not mean there are no known or expected pathogenic variants in that gene, or other variants that may confer susceptibility to the disorders listed.

Resources

MyGene2 is a portal through which families with rare genetic conditions who are interested in sharing their health and genetic information can connect with other families, clinicians, and researchers. If you are interested in learning more and/or participating, please visit www.mygene2.org.

[redacted]

DOB
[redacted]/2011

Accession
1590453

REVISED REPORT

Submitter Patient ID(s) [redacted], L16-109R28

GenomeConnect is an NIH initiative created to enable individuals and families with the same genetic variant or medical history to connect and share de-identified information. If you are interested in participating, please visit www.genomeconnect.org.

Additional Samples Submitted

Sample(s) submitted for analysis by next generation sequencing: mother (GeneDx #1590466), father (GeneDx #1590480).

Quality Metrics

Mean Depth of Coverage[1]	164x	Quality threshold[2]	97.2%

The above values represent metrics from this test evaluation. [1]Mean depth of coverage refers to the mean number of sequence reads obtained across the whole exome; specifically the coding exons and splice junctions of protein-coding RefSeq genes that are captured by massively parallel (NextGen) sequencing. [2]The quality threshold refers to the percentage of the test, which is covered by at least 10 sequence reads (10x coverage). This is the analysis threshold level that permits high quality exome variant base calling, annotation and evaluation. Average quality thresholds may range from >90-95% of the targeted region, indicating a small portion of the target region may not be covered with sufficient depth or quality to confidently call variant positions.

Copy Number Variant (CNV) Detection

CNV analysis was performed using the data. Unless reported above, there is no indication of a clinically relevant deletion or duplication of three or more exons in the data for this proband.

Methods

Previously generated exome sequence data from this individual's genomic DNA sample and additional familial specimens (if submitted) was re-annotated and re-analyzed in comparison with the published human genome build GRCh37/UCSC hg19. The targeted coding exons and splice junctions of the known protein-coding RefSeq genes and the average depth of coverage and data quality threshold values remain the same from the initial analysis. Using a custom-developed analysis tool (XomeAnalyzer), data were filtered and analyzed to identify sequence variants and most deletions and duplications involving three or more coding exons (Retterer et al., 2015). Smaller deletions or duplications may not be reliably identified. Reported clinically significant variants were confirmed by an appropriate orthogonal method in the proband and, if submitted, in selected relatives as necessary. Sequence and copy number variants are reported according to the Human Genome Variation Society (HGVS) or International System for Human Cytogenetic Nomenclature (ISCN) guidelines, respectively. Reportable variants include pathogenic variants, likely pathogenic variants and variants of uncertain significance. Likely benign and benign variants, if present, are not routinely reported.

Disclaimer

Genetic testing using the methods applied at GeneDx is expected to be highly accurate. The absence of definitive pathogenic findings does not rule out the diagnosis of a genetic disorder as some genetic abnormalities may be undetectable with this test. It is possible that a disease-causing variant may be located in a genomic region that is not sufficiently sequenced using the current technologies and, therefore, would not be detected. Additionally, multifactorial disorders and genetic disorders due to nucleotide repeat expansion/contraction, abnormal DNA methylation, and other mechanisms may not be detectable with this test. This test also may not reliably detect mosaicism, balanced chromosomal aberrations, and partial-exon deletions/insertions of 20 bp or more. Regions of certain genes have inherent sequence properties (for example: repeat, homology, or pseudogene regions, high GC content, rare polymorphisms) that yield suboptimal data, potentially impairing accuracy of the results. False negative results may also occur in the setting of bone marrow transplantation, recent blood transfusion, or suboptimal DNA quality. The chance of a false positive or false negative result due to laboratory errors incurred during any phase of testing cannot be completely excluded. Interpretations are made with the assumption that any clinical information provided, including family relationships, is accurate. Consultation with a genetics professional is recommended for interpretation of results.

This test was developed and its performance characteristics determined by GeneDx. This test has not been cleared or approved by the U.S. Food and Drug Administration. The FDA has determined that such clearance or approval is not necessary. The test is used for clinical purposes and should not be regarded as investigational or for research. The laboratory is certified under the Clinical Laboratory

Improvement Amendments of 1988 (CLIA) as qualified to perform high-complexity clinical testing.

References

Lek et al. (2016) Nature 536 (7616):285-91 (PMID: 27535533); Miller DT et al. (2021) Genet Med (PMID: 34012068); Kalia et al. (2017) Genet. Med.19 (2):249-255 (PMID: 27854360); Green et al. (2013) Genet. Med.15 (7):565-74 (PMID: 23788249); Richards et al. (2015) Genet. Med.17 (5):405-24 (PMID: 25741868); Retterer et al. (2015) Genet. Med. 17 (8):623-9 (PMID: 25356966); Iossifov I et al. (2014) Nature 515 (7526):216-21 (PMID: 25363768); Mohlmann S et al. (2014) Acta Crystallogr D Biol Crystallogr 70 (Pt 6):1622-30 (PMID: 24914973); Deciphering Developmental Disorders Study. (2017) Nature 542 (7642):433-438 (PMID: 28135719); Burns W et al. (2021) Am J Med Genet A (PMID: 34050707);

Report Electronically Signed By

Amber H. Begtrup PhD, FACMG
Director, Clinical Genetics

[redacted]

DOB
[redacted]/2011

Accession
1590453

REVISED REPORT

Submitter Patient ID(s) [redacted], L16-109R28

Variant Table

**Gene: Coding DNA**	**DDX23: c.1582 C>T**
**Variant (Protein)**	**p.(R528C) (p.(R528C))**
**Classification**	Pathogenic Variant
**Zygosity**	Heterozygous
**Chr: Position**	12: 49227281
**dbSNP**	
**gnomAD_Freq**	
**gnomAD_AMR**	
**gnomAD_NFE**	
**gnomAD_AFR**	
**gnomAD_EAS**	
**gnomAD_FIN**	
**gnomAD_Other**	
**gnomAD_SAS**	
**gnomAD_ASJ**	
**gnomAD_Hom**	
**Provean**	-7.68 (D)
**ClinVar**	Uncertain significance

This supplement provides evidence to support the classification of each reportable variant in the attached result report. This information is provided as a resource. It is not inclusive of all available information used by GeneDx for variant classification, and individual data elements may be weighted differently to derive at the classification. This information is subject to change and may differ from what is currently available. Results should always be interpreted in the context of the patient's clinical presentation. Blank fields indicate that no data were available at time of analysis.

- dbSNP - NCBI repository for single base nucleotide substitutions and short deletion and insertion polymorphisms https://www.ncbi.nlm.nih.gov/snp/
- The Genome Aggregation Database (gnomAD) combines exome and genome sequencing data from a variety of large-scale sequencing projects, including approximately 15,000 genomes and 123,000 exomes, including individuals recruited for disease-specific studies such as cancer and cardiovascular diseases. (PMID 32461654).
- gnomAD_Freq - variant allele frequency (in percent) from approximately 15,000 genomes and 123,000 exomes in the Genome Aggregation Database. Select ancestries include: gnomAD_AMR (Admixed American/Latino); gnomAD_AFR (African); gnomAD_EAS (East Asian); gnomAD_FIN (Finnish of European ancestry); gnomAD_NFE (non-Finnish of European ancestry); gnomAD_SAS (South Asian); gnomAD_ASJ (Ashkenazi Jewish). gnomAD_Hom - number of individuals homozygous for the variant.
- gnomAD_AMR- variant frequency (in percent) for individuals of Latino ancestry
- PROVEAN (Protein Variation Effect Analyzer) - predicts whether an amino acid substitution or indel affects the biological function of a protein using a delta alignment score from -14 to +14 (< or = -2.5, predicted deleterious; >-2.5, predicted neutral).
- Other published in silico algorithms, including those that predict splicing impact, may be considered for variant analysis. In silico scores may change. In silico models use algorithms that predict the effect a variant may have on the protein. Thus, predictions should be interpreted with caution and only be used in combination with other available evidence to support the classification of any variant (PMID 23056405).
- ClinVar - Classification of variant in ClinVar database, an NCBI archive of human variants with supporting evidence of phenotypic association. https://www.ncbi.nlm.nih.gov/clinvar/ (PMID 26582918).

Appendix 1. ACMG SF v2.0 (September, 2016) (Kalia et al., 2017):

Gene	Disease	Mode of Inheritance	MIM-Gene
ACTA2	Marfan Syndrome; Loeys-Dietz Syndromes; TAAD	Autosomal Dominant	102620
ACTC1	Hypertrophic cardiomyopathy; Dilated cardiomyopathy	Autosomal Dominant	102540
APC	Familial adenomatous polyposis	Autosomal Dominant	611731
APOB	Familial hypercholesterolemia	Autosomal Dominant	107730
ATP7B	Wilson disease	Autosomal Recessive	606882
BMPR1A	Juvenile polyposis	Autosomal Dominant	601299
BRCA1	Hereditary Breast and Ovarian Cancer	Autosomal Dominant	113705
BRCA2	Hereditary Breast and Ovarian Cancer	Autosomal Dominant	600185
CACNA1S	Malignant hyperthermia susceptibility	Autosomal Dominant	114208
COL3A1	Ehlers-Danlos syndrome – vascular type	Autosomal Dominant	120180
DSC2	Arrhythmogenic right ventricular cardiomyopathy	Autosomal Dominant	125645
DSG2	Arrhythmogenic right ventricular cardiomyopathy	Autosomal Dominant	125671
DSP	Arrhythmogenic right ventricular cardiomyopathy	Autosomal Dominant	125647
FBN1	Marfan Syndrome; Loeys-Dietz Syndromes; TAAD	Autosomal Dominant	134797
GLA	Hypertrophic cardiomyopathy	X-linked	300644
KCNH2	Long QT syndrome	Autosomal Dominant	152427
KCNQ1	Long QT syndrome	Autosomal Dominant	607542
LDLR	Familial hypercholesterolemia	Autosomal Dominant	606945
LMNA	Dilated cardiomyopathy; Arrhythmogenic right ventricular cardiomyopathy	Autosomal Dominant	150330
MEN1	Multiple Endocrine Neoplasia Type 1	Autosomal Dominant	613733
MLH1	Lynch Syndrome	Autosomal Dominant	120436
MSH2	Lynch Syndrome	Autosomal Dominant	609309
MSH6	Lynch Syndrome	Autosomal Dominant	600678
MUTYH	MYH-Associated Polyposis	Autosomal Recessive	604933
MYBPC3	Hypertrophic cardiomyopathy; Dilated cardiomyopathy	Autosomal Dominant	600958
MYL2	Hypertrophic cardiomyopathy	Autosomal Dominant	160781
MYL3	Hypertrophic cardiomyopathy	Autosomal Dominant	160790
MYH7	Hypertrophic cardiomyopathy; Dilated cardiomyopathy	Autosomal Dominant	160760
MYH11	Marfan Syndrome; Loeys-Dietz Syndromes; TAAD	Autosomal Dominant	160745
NF2	Neurofibromatosis type 2	Autosomal Dominant	607379
OTC	Ornithine transcarbamylase deficiency	X-Linked	300461
PCSK9	Familial hypercholesterolemia	Autosomal Dominant	607786
PKP2	Arrhythmogenic right ventricular cardiomyopathy	Autosomal Dominant	602861
PMS2	Lynch Syndrome	Autosomal Dominant	600259
PRKAG2	Hypertrophic cardiomyopathy; Dilated cardiomyopathy	Autosomal Dominant	602743
PTEN	PTEN Hamartoma Tumor Syndrome	Autosomal Dominant	601728
RB1	Retinoblastoma	Autosomal Dominant	614041
RET	Multiple Endocrine Neoplasia type 2;<br>Familial Medullary Thyroid Cancer	Autosomal Dominant	164761
RYR1	Malignant hyperthermia susceptibility	Autosomal Dominant	180901
RYR2	Catecholaminergic polymorphic ventricular tachycardia	Autosomal Dominant	180902
SCN5A	Long QT syndrome; Brugada syndrome; Dilated Cardiomyopathy	Autosomal Dominant	600163
SDHAF2	Hereditary Paraganglioma-Pheochromocytoma Syndrome	Autosomal Dominant	613019
SDHB	Hereditary Paraganglioma-Pheochromocytoma Syndrome	Autosomal Dominant	185470
SDHC	Hereditary Paraganglioma-Pheochromocytoma Syndrome	Autosomal Dominant	602413
SDHD	Hereditary Paraganglioma-Pheochromocytoma Syndrome	Autosomal Dominant	602690
SMAD3	Marfan Syndrome; Loeys-Dietz Syndromes; TAAD	Autosomal Dominant	603109
SMAD4	Juvenile polyposis	Autosomal Dominant	600993
STK11	Peutz-Jeghers syndrome	Autosomal Dominant	602216
TGFBR1	Marfan Syndrome; Loeys-Dietz Syndromes; TAAD	Autosomal Dominant	190181
TGFBR2	Marfan Syndrome; Loeys-Dietz Syndromes; TAAD	Autosomal Dominant	190182
TMEM43	Arrhythmogenic right ventricular cardiomyopathy	Autosomal Dominant	612048
TNNI3	Hypertrophic cardiomyopathy; Dilated cardiomyopathy	Autosomal Dominant	191044
TNNT2	Hypertrophic cardiomyopathy; Dilated cardiomyopathy	Autosomal Dominant	191045
TP53	Li-Fraumeni Syndrome	Autosomal Dominant	191170
TPM1	Hypertrophic cardiomyopathy; Dilated cardiomyopathy	Autosomal Dominant	191010
TSC1	Tuberous Sclerosis Complex	Autosomal Dominant	605284
TSC2	Tuberous Sclerosis Complex	Autosomal Dominant	191092
VHL	Von Hippel Lindau syndrome	Autosomal Dominant	608537
WT1	WT1-related Wilms tumor	Autosomal Dominant	607102
