# Supplementary material for: DDX23 gain-of-function linked to autism spectrum disorder with intellectual disability, motor delay, and seizure
Source: Genet Med Open. 2026 Jul 5;4:104484. doi: 10.1016/j.gimo.2026.104484 (PMC13507733; doi:10.1016/j.gimo.2026.104484)
Supplement: Supplementary file 3 [file mmc3.pdf]

[redacted]

GeneDx

DOB [redacted]

Accession [redacted]

Submitter Patient ID(s) [redacted]

**Sample**
Source: OraCollect Buccal
Date Collected: 03-APR-2023
Date Received: 05-APR-2023

**Testing**
Date Started: 07-APR-2023
Date Reported: 11-MAY-2023

**Provider**
Account #: NX011
VARGAS, MD, MARCELO

## Test(s) Requested

Diagnostic Testing / XomeDx / Clinical Exome Sequence Analysis

## Clinical Indication

Individual with growth delays, nail hypoplasia, hypotonia, bilateral sensorineural hearing loss, and global developmental delay.

**Result: Positive**

- Causative variant(s) in disease genes associated with reported phenotype: **POSITIVE**
- Variant(s) in disease genes possibly associated with reported phenotype: **None Identified**
- ACMG Secondary Findings: **None Identified**

## Causative Variant(s) in Disease Genes Associated with Reported Phenotype:

| Gene | Disease | Mode of Inheritance | Variant | Zygosity | Inherited From | Classification |
|---|---|---|---|---|---|---|
| DDX23 | DDX23-related neurodevelopmental disorder | Autosomal Dominant | c.1583 G>A p.(R528H) | Heterozygous | De Novo | Pathogenic Variant |

## Interpretation

**This individual is heterozygous for a pathogenic variant in the DDX23 gene, which is consistent with DDX23-related neurodevelopmental disorder. As both parents tested negative for the variant, most likely it arose de novo in the child; however, the possibility of germline mosaicism must be considered.**

## Recommendation(s)

- Genetic counseling is recommended to discuss the implications of these results.
- Correlation of these findings with the clinical features of this individual is recommended.
- Though neither parent has the pathogenic variant, the possibility of germline mosaicism cannot be excluded and offspring of either parent may be at risk to inherit this variant.
- Molecular prenatal diagnosis may be considered for the pathogenic variant.
- Please contact GeneDx to discuss testing requirements prior to submitting fetal samples.

GeneDx

## DDX23

**GENE SUMMARY**

The DDX23 gene is a member of the DEAD box family of RNA helicases and plays a role in the formation of an active spliceosome and pre-mRNA splicing (PMID: 24914973). De novo heterozygous missense variants have been reported in individuals with features including global developmental delay, autism spectrum disorder, seizures, hypotonia, short stature, low weight, microcephaly, and dysmorphic facial features (PMID: 34050707).

For this gene, 100% of the coding region was covered at a minimum of 10x by this test. There is no indication of a multi-exon deletion/duplication involving this gene in the sequencing data.

p.(Arg528His) (CGT>CAT): c.1583 G>A in exon 13 of the DDX23 gene (NM_004818.2)

The proband's mother[redacted]and father[redacted]do not harbor the p.(R528H) variant in the DDX23 gene.

- Identified as a de novo variant in the published literature (Turner et al., 2019) and as an apparently de novo variant in patients in the published literature (Burns et al., 2021) with DDX23-related neurodevelopmental disorder
- A different missense change at this residue (R528C) has been reported as pathogenic in the published literature in association with DDX23-related neurodevelopmental disorder (Burns et al., 2021)
- Identified as a de novo variant with confirmed parentage in this patient with clinical features consistent with DDX23-related neurodevelopmental disorder
- In silico analysis supports that this missense variant has a deleterious effect on protein structure/function
- Not observed at significant frequency in large population cohorts (gnomAD)

**We interpret this as a Pathogenic Variant.**

## Additional Analysis Comments

The analysis for the proband includes evaluation of variants that are identified to be de novo (when both parents submitted), compound heterozygous (when both parents submitted), homozygous, heterozygous and X-linked. In addition, analysis takes into consideration family structure, reported phenotype, and provided clinical and/or differential diagnosis. This is a phenotype-driven test of a very large number of genes. Therefore, reported results are focused on pathogenic and likely pathogenic variants in genes related to the phenotype of the proband, while variants of uncertain significance are only rarely reported at our discretion. Sequence variants are classified based on the ACMG/AMP guidelines (PMID: 25741868). Updates to the classification of a sequence variant may be accessed through ClinVar (www.clinvar.com). Independent analysis of submitted relative samples is not performed unless ordered separately.

## ACMG Secondary Findings Limitations

Pathogenic and/or likely pathogenic variants in the genes recommended by the ACMG SF v3.1 are reported for the proband, in accordance with the reporting structure recommended by the ACMG (PMID: 35802134). Only pathogenic and likely pathogenic variants associated with the disorders and inheritance specified by the ACMG are reported (PMID: 34012069, 25741868). Reporting the presence or absence of the proband's identified secondary findings is available for relatives who underwent analysis as part of the proband's test. ACMG secondary findings are not reported for relatives that opted out of receiving ACMG secondary findings. Variants that may be present in a relative, but are not present in the proband, would not be detected and therefore are not reported. The absence of reportable secondary findings for any particular gene does not mean there are no pathogenic, likely pathogenic, or other variants in that gene that may confer susceptibility to the associated disorder.

## Resources

MyGene2 is a portal through which families with rare genetic conditions who are interested in sharing their health and genetic information can connect with other families, clinicians, and researchers. If you are interested in learning more and/or participating, please visit
